# Intravascular Large B Cell Lymphoma as a Rare Cause of Reversed Halo Sign: A Case Report

**DOI:** 10.1097/MD.0000000000003138

**Published:** 2016-03-25

**Authors:** Min Peng, Juhong Shi, Hongrui Liu, Guangxi Li

**Affiliations:** From the Department of Respiratory Medicine (MP, JS); Department of Pathology (HL), Peking Union Medical College Hospital, Chinese Academy of Medical Sciences and Peking Union Medical College, Beijing, China; and Department of Medicine, Division of Integrative Medicine, Mayo Clinic, Rochester, MN (GL).

## Abstract

Intravascular large B cell lymphoma (IVLBCL) is a rare type of extranodal diffused large B-cell lymphoma. IVLBCL with primary lung lesion is very rare and it is very difficult to diagnose. Radiographic findings of pulmonary IVLBCL are nonspecific and resembling interstitial lung diseases. Reversed halo sign (RHS) was initially reported in patients diagnosed with cryptogenic organizing pneumonia and then described in a variety of diseases with inflammatory, infectious, autoimmune, and malignant causes. This is the first case of IVLBCL that has presented with RHSs on CT scan.

A 59-year-old Chinese man presented with a 4-month history of a nonproductive cough and a weight loss of 5 kg. Physical examination was unremarkable. High-resolution computed tomography scan of the chest showed bilateral patchy ground glass opacities (GGOs) and RHSs. Laboratory tests were unremarkable except elevated serum lactate dehydrogenase (LDH). Surgical lung biopsy was performed. Light microscopic examination of the specimen disclosed diffuse alveolar septal widening caused by neoplastic lymphocytes, which were positive for CD20 and infiltrated in the alveolar capillaries. The patient was diagnosed with IVLBCL and underwent chemotherapy and autologous blood stem cell transplantation. The patient is still alive 5 years after diagnosis.

IVLBCL is a rare cause of RHS and should be considered in differential diagnosis of RHS. An increased serum LDH concentration is another important clue.

## INTRODUCTION

Intravascular large B-cell lymphoma (IVLBCL) is a rare disease entity of malignant lymphoma, characterized by the involvement of lymphoma cells in the lumina of vessels in various organs.^1^ The disease most commonly affects the skin and central nervous system but may involve other organs.^1^ Although lung involvement can be demonstrated on autopsy in approximately 60% of cases,^2^ a primary presentation of IVLBCL in the lungs is uncommon. The majority of patients with lung involvement had signs and radiologic findings that resembled interstitial lung disease.^3–8^ The diagnosis of pulmonary IVLBCL is frequently difficult to make because the clinical and radiographic findings are often nonspecific.

The reversed halo sign (RHS) is defined as a rounded area of ground-glass opacity surrounded by a complete or nearly complete ring of consolidation on high-resolution CT (HRCT) scan of the chest.^9^ RHS was initially reported in cryptogenic organizing pneumonia and then was described in a wide variety of diseases with infectious, inflammatory, or autoimmune and malignant causes.^9^ But RHSs in intravascular lymphoma have not been reported before.

Herein, we report a patient with IVLBCL presenting primarily in the lungs with GGOs and RHSs on HRCT.

### Case Report

A 59-year-old man presented with a 4-month history of a nonproductive cough and a weight loss of 5 kg (timeline shown in Table 1). There was no fever, dyspnea, or hemoptysis. He had received a diagnosis of paroxysmal atrial fibrillation 12 years before. A radiofrequency ablation had been performed 3 years ago, and amiodarone had been prescribed for half a year. He was not a smoker and had no known exposure to chemicals, fumes, or dusts.

Upon physical examination, the patient was afebrile and well nourished. There was no evidence of lymphadenopathy, skin lesions, dementia, or focal neurologic deficit. His lungs were clear on auscultation. The abdomen was nontender, without a palpable mass, or hepatosplenomegaly.

Routine blood tests, liver and renal function assays, the erythrocyte sedimentation rate, and screening tests for rheumatological disease were all within normal ranges, although the level of serum lactate dehydrogenase (LDH) was elevated at 712 U/L (normal range 110–240 U/L). The pulmonary function test and arterial blood analysis were normal.

**FIGURE 1:**
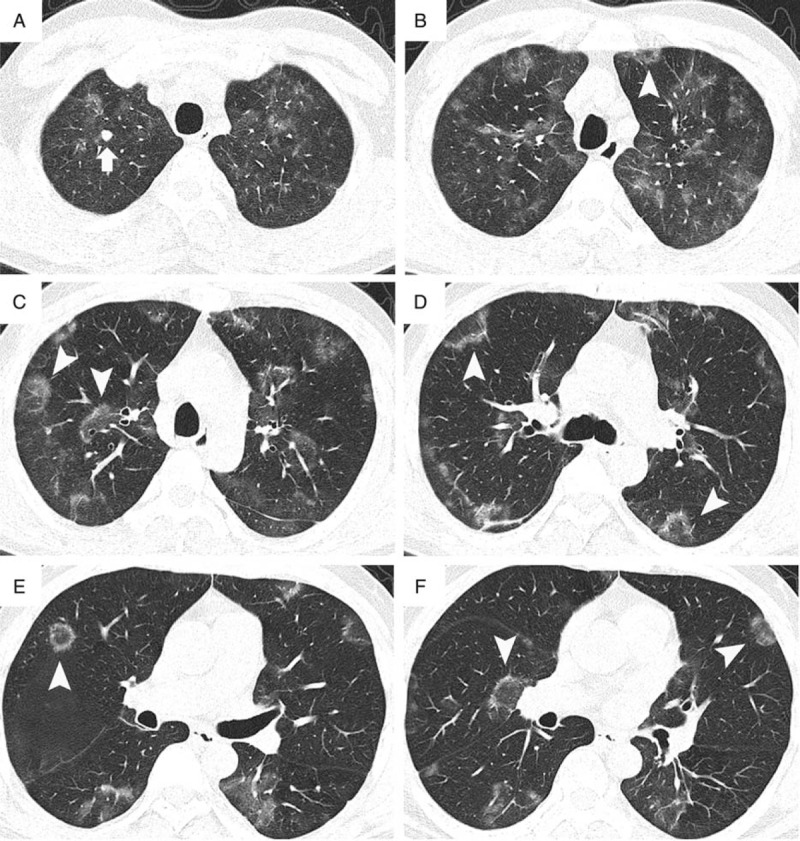
Imaging of the lung. (A) High-resolution computed tomography scans demonstrate multiple ground-glass opacities bilaterally in the uppers lung and a small nodule (arrow) in the right upper lung. (B–F) High-resolution computed tomography scans demonstrate bilateral patchy ground-glass opacities, multiple lesions of the reversed halo sign (arrowhead).

The chest radiograph revealed patchy high-attenuation opacities predominantly in the upper lungs. A high-resolution computed tomography (HRCT) scan of the chest showed bilateral patchy ground-glass opacities (GGOs) and RHSs predominantly in the upper lungs (Figure 1A-F). There was a small nodule in the right upper lung (Figure 1A). No enlarged lymph nodes or pleural effusions were identified. In the nonenhanced CT scan of the abdomen that was performed simultaneously, there was no evidence of lymphadenopathy or splenomegaly.

A bronchoscopy was performed and revealed no endobronchial lesions. The bronchoalveolar lavage that was performed in the right middle lobe was negative for acid-fast bacilli, fungi, or malignant cells. The cell analysis of the bronchoalveolar lavage liquid revealed that the lymphocyte differential count was 75% and that the CD4/CD8 ratio was 0.4. The transbronchial lung biopsy specimen from the right lower lobe revealed evidence of chronic inflammation.

The clinical differential diagnosis included chronic infections, interstitial lung disease and malignant disease. However, the patient lacked a fever, had a cough that did not produce of yellow sputum, and had a bronchoalveolar lavage that was negative on microbiologic examination. There were no laboratory tests or a history that suggested interstitial lung disease secondary to collagen vascular disease or environmental exposure. Further, there was no evidence of malignancy in the bronchoalveolar lavage specimens or the transbronchial lung biopsy.

**FIGURE 2:**
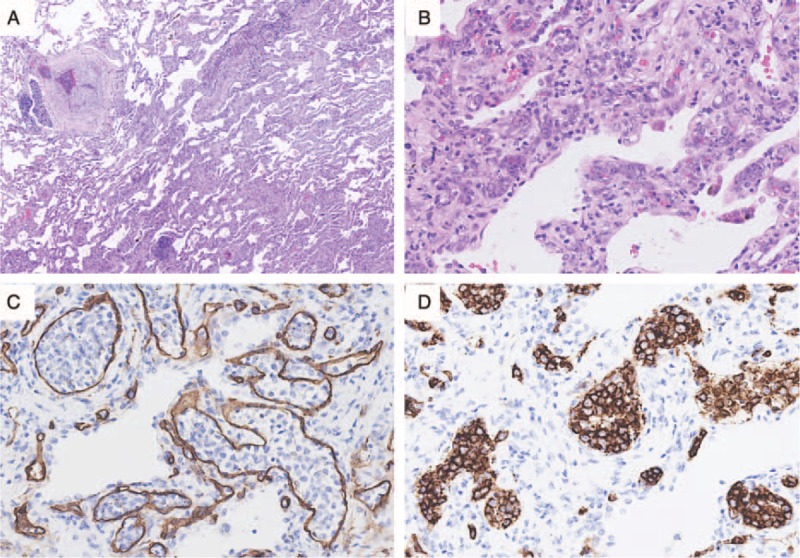
Histology of the lung. (A) Alveolar septa were widened (hematoxylin and eosin stain, magnification ×100). (B) Large lymphoid cells infiltrated the alveolar septa (hematoxylin and eosin stain, magnification ×200). (C) Neoplastic lymphocytes were located in the alveolar capillaries (immunohistochemical stain for CD34, magnification ×400). (D) Intravascular neoplastic large B cells were detected (immunohistochemical stain for CD20, magnification ×400).

A video-assisted thoracoscopic biopsy of the left lung was performed. Light microscopic examination of the specimen disclosed diffuse alveolar septal widening caused by large lymphoid cells infiltrating the alveolar septa, whereas the alveolar space was not involved (Figure 2A, B). Immunohistochemical staining for CD34 demonstrated that lymphoid cells had accumulated exclusively in the lumens of the pulmonary capillaries, venules, and pulmonary arterioles (Figure 2C). Further immunohistochemical staining showed that the intravascular lymphoid cells were positive for the B-cell marker CD20 (Figure 2D).

The patient was diagnosed with IVLBCL. Positron emission tomography-computed tomography (PET-CT) scans performed after the lung biopsy showed increased metabolic activity in the lungs, the left adrenal gland, and the 12th thoracic vertebra, suggesting stage IV of IVLBCL. The PET-CT also revealed multiple new nodules in the lungs, suggesting disease progression. The patient underwent 5 courses of chemotherapy (R-CHOP plus Methotrexate). After 2 months of treatment, a repeat CT scan showed resolution of the lung lesions, and the serum LDH was in the normal range. Patient was transferred to another hospital and underwent autologous peripheral blood stem cell transplantation. After that the patient received several courses of retuximab and cytokine-induced killer cell immunotherapy. The patient is stable 5 years after diagnosis (Table 1).

**TABLE 1:**
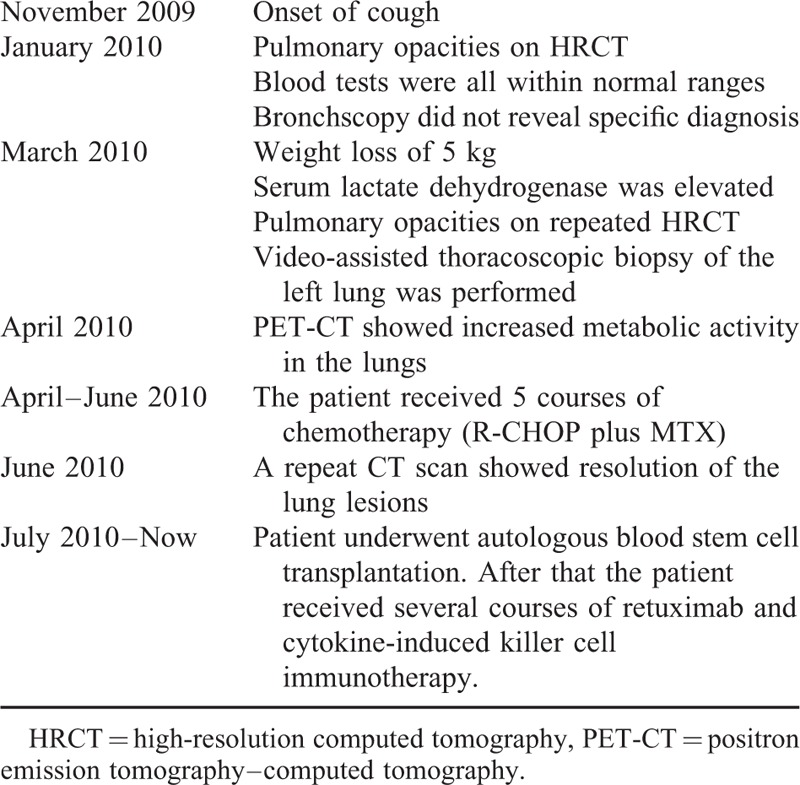
Timeline

## DISCUSSION

IVLBCL is characterized by the involvement of lymphoma cells in the lumina of vessels in various organs. According to the current WHO classification, IVLBCL is defined as an extranodal B-cell lymphoma.^1^ Most intravascular lymphoid cells express B-cell-associated antigens, although intravascular T-cell or natural killer cell lymphomas have been reported occasionally.^1^ The disease most commonly affects the skin and central nervous system but may involve other organs, such as the lungs, liver, kidneys, adrenal glands, and prostate.^1^ The most common clinical manifestations are fever; neurologic abnormalities, including dementia or focal defects; and cutaneous nodules or plaques.^1^

Although lung involvement can be demonstrated on autopsy in approximately 60% of cases,^2^ a primary presentation of IVLBCL in the lungs is uncommon. The diagnosis of pulmonary IVLBCL is frequently difficult to make because the clinical and radiographic findings are often nonspecific, especially when skin lesions or neurological abnormalities are absent.

The clinical presentations of pulmonary intravascular lymphoma are nonspecific. The majority of patients with lung involvement had signs and symptoms that resembled interstitial lung disease.^3–8^ In a few cases, IVLBCL manifested as pulmonary hypertension^2,10^ and pulmonary embolism or infarction.^11–13^ The common symptoms include dyspnea, cough, fever, fatigue, night sweats, and weight loss. Usually, the coughs were nonproductive.^3,7,8,13–15^ Dyspnea, which usually occurred on exertion, was the prominent symptom.^2–4,6,10–12,14–16^ Dyspnea developed progressively, though occasionally might have had an acute onset. Hypoxemia was common.^2,3,5,10–12,14–16^ Hypoxemia may be severely out of proportion with the imaging findings^15^ and may be improved by treatment.^17^ In this patient, the only symptoms were a nonproductive cough and weight loss; other organ involvement, especially the skin and neurological system, was absent. The clinical manifestations did not readily suggest the diagnosis.

The most commonly reported laboratory finding was an elevated level of serum LDH.^5,10,13–15,18,19^ In this patient, serum LDH was elevated and back to the normal range after chemotherapy. Thus, an increased serum LDH concentration is an important clue suggesting intravascular lymphoma in patients with interstitial lung disease on HRCT.

Pulmonary IVLBCL, a rare type of malignant lymphoma, has radiological features that are different from those of the common lymphomas, which usually manifest as solitary or multiple pulmonary nodules, a mass or consolidation, or intrathoracic lymphadenopathy on CT scan. Chest radiography of individuals with pulmonary IVLBCL may be unremarkable or may show interstitial infiltrates.^3–6,8^ The CT findings have been described as patchy, subpleural, or diffused GGOs,^5–8,12,13,15,17^ sometimes with intralobular septal thickening,^8^ interlobular septal thichening,^6^ linear shadows,^13^ or reticulonodular shadows.^7^ Some other uncommon CT findings have been reported, such as bilateral centrilobular ground-glass nodules and peribronchial GGOs,^19^ localized or segmental consolidation,^8,16^ thickening of bronchovascular bundles,^16^ air trapping,^14^ and subpleural wedge-shaped opacities.^5,12,13^

In this patient, the HRCT scans showed diffused GGOs, which were similar to those in previous reports.^5–8,12,13,15^ However, another major finding on HRCT was a central GGO surrounded by a ring of consolidation, termed RHS, which had not been reported in previous cases. The RHS was initially considered to be specific to cryptogenic organizing pneumonia. Subsequently, various authors have demonstrated the presence of RHS in a wide spectrum of diseases, including infectious diseases (paracoccidioidomycosis, TB, zygomycosis, invasive pulmonary aspergillosis, Pneumocystis jiroveci pneumonia, histoplasmosis, cryptococcosis), and noninfectious diseases (sarcoidosis, edema, lepidic predominant adenocarcinoma, granulomatosis with polyangiitis).^9^ RHS was also reported in patients with pulmonary embolism or infarction.^20^ Although pulmonary embolisms and infarctions have been reported in other patients with IVLBCL,^11–13^ in this patient, pathological findings did not reveal any evidence of pulmonary infarction. To our knowledge, this is the first case of IVLBCL that has presented with RHSs on CT scan. Based on previous reports, RHS is a relatively nonspecific sign. IVLBCL is a rare cause of RHS.

Pathologically, intravascular lymphomas are characterized by neoplastic lymphoid cells that exist mainly in the lumina of the small or intermediate-sized vessels in various organs.^1^ In the case of individuals with pulmonary IVLBCL, histological examination revealed that lymphoma cells accumulate in the small- or intermediate-sized vessels of the pulmonary interstitial tissues and make the alveolar septa widen diffusely, whereas the alveolar spaces are almost intact.^7,8,18^

The accumulation of lymphoid cells in the lumen of vessels is the distinct pathological feature of IVLBCL, which makes the cases of pulmonary IVLBCL unique in the clinical and radiological manifestations. Typically, because of diffuse pulmonary vessel obliteration, dyspnea and hypoxemia are common and might even be out of proportion to lesions seen on CT scans. Some cases have manifested as pulmonary hypertension^2,10^ or pulmonary embolism.^11^ Because the alveolar septa are thickened by the diffuse infiltration of neoplastic lymphocytes in the interstitial vessels, the chest CT findings of pulmonary IVLBCL show multiple or diffuse GGOs or reticular opacities, which usually resemble interstitial lung disease.^3–8^ In this patient, the RHSs on CT might also correspond to interstitial thickening. There were no evidence of bronchiolitis obliterans organizing pneumonia, granulomatosis, infarction or hemorrhage, as reported in previous studies.^9,20^

## CONCLUSIONS

In summary, we describe a patient with IVLBCL presenting primarily in the lungs with GGOs and RHSs on HRCT. Pulmonary IVLBCL, a rare and unique form of lymphoma, is difficult to diagnose because it has nonspecific clinical and radiological features. It is important for the pulmonologist to recognize the possibility of this unusual lymphoma when a CT scan shows interstitial opacities with RHSs. An increased serum LDH concentration is another important clue.

## CONSENT

This study was approved by Peking Union Medical College Hospital Institutional Review Board (Reference number for ethics approval: 2015–6–126). Informed consent for publication of the clinical information was obtained from the patient when he was admitted to the hospital. A copy of IRB is available for review by the Editor of this journal.
